# Characterization of experimental cerebral malaria by volumetric MRI A comparative study across the sexes

**DOI:** 10.1371/journal.pone.0328693

**Published:** 2025-08-18

**Authors:** Alicia Comino Garcia Muñoz, Oumaima Marfouk, Constance P. Michel, Isabelle Varlet, Emilien Royer, Teodora-Adriana Perles-Barbacaru, Angèle Viola

**Affiliations:** Faculté des Sciences Médicales et Paramédicales la Timone, Aix-Marseille Université, CNRS, Centre de Résonance Magnétique Biologique et Médicale, UMR 7339, Marseille, France; Kaohsuing Medical University Hospital, TAIWAN

## Abstract

Cerebral malaria (CM), a potentially lethal neurological complication of the infection by *Plasmodium falciparum*, affects mostly the pediatric population under 5 years old in sub-Saharan Africa. This clinical syndrome is characterized on anatomical brain imaging by microhemorrhages, parenchymal lesions and brain edema. Epidemiological studies based on sex or gender are rare and do not allow to draw any conclusions on a possible sexual dimorphism in CM. However, some regional data and genetic studies suggest a possible influence of sex on the susceptibility to this clinical syndrome and complications in surviving patients. The murine model of experimental cerebral malaria (ECM) in mice has proven to be a useful and reliable tool to study the pathogenic mechanisms and possible therapeutical approaches for CM. In this study, we used *in vivo* magnetic resonance imaging (MRI) to assess the differences linked to sex in the development of experimental CM in C57BL/6J mice infected with the murine parasite *Plasmodium berghei* ANKA. Our volumetric analysis reveals sex-dependent differences in brain swelling and lesion distribution, particularly microhemorrhages, as well as a regionalization of brain edema in ECM, with swelling-prone structures common to both sexes like the cortex and the pons, and others which show sex-specific alterations like the inferior and superior colliculi or the midbrain. Together, our results indicate that ECM is more severe in male than in female C57Bl/6J mice.

## Introduction

Malaria, a major contributor to the global burden of infectious diseases, accounted for 263 million cases in 2023 and 597 000 deaths [1]. Cerebral malaria (CM) is a complication that occurs in 1–2% of *Plasmodium falciparum* infections, children under the age of 5 years in sub-Saharan Africa being the most affected [2,3]. The fatality rate of this acute encephalopathy is 15–20% with anti-malaria treatments and 100% when untreated [4].

Pediatric CM is characterized by seizures, altered consciousness and coma with brain swelling being a major predictor of death [5,6]. In adults, CM occurs as part of a multi-organ failure [5], and the mortality is higher than in children [7].

The existence of sex differences in CM is not clear-cut. Data on incidence and mortality are generally not stratified by sex or gender and studies on sex-related pathogenesis are still rare [8,9]. Khadanga et al. (2014) reported a higher mortality and incidence of convulsions in non-pregnant women than in men, whereas Ikome et al. (2002) reported no correlation between the prevalence of CM and sex [10] However, Wilson et al. (2013) demonstrated that males with a polymorphism in the gene promoter of CXCL10, a pro-inflammatory chemokine, had an increased risk for CM [11]. Male sex has recently been included as a risk factor for neurological sequelae after CM [12].

Experimental CM (ECM) in mice is a clinically relevant model [13,14]. CM-susceptible strains infected with *Plasmodium berghei* ANKA (PbA) develop a neurological syndrome characterized by seizures, ataxia coma and ultimately death [14]. Both human and murine CM present early sequestration and adhesion of parasitized red blood cells (pRBC) to the brain microvascular endothelium [15] resulting in endothelial activation and inflammatory damage [15–17]. Among the mouse strains susceptible to CM [18,19] CBA and C57BL/6 mice are probably the most studied. In both strains, CM develops in 6–7 days and the percentage of survival is generally less than 10% 10 days after inoculation. However, CBA and C57BL/6 strains show differences in gene expression profiles during ECM. In addition, cerebral levels of TNF and lymphotoxin α, leucocyte recruitment (in particular CD4^+^T cells) in brain and the cytokine profiles in plasma vary between the two strains [20]. TNF is the main cytokine involved in ECM in CBA mice, while the main modulator of ECM in C57BL/6 mice is lymphotoxin α [21,22]. Compared to the C57BL/6 strain, CBA mice are considered to have higher immunological activity in brain during ECM [20]. Despite these differences, the two strains present a similar disease course with no difference in parasitemia in female mice [23] and similar clinical and neurological manifestations.

Few studies have reported sex differences in ECM. It has been shown that males of the susceptible strain LG/J and intermediately susceptible strain MRL/MpJ have a lower survival than females [19], and that CBA/Ca males present a more severe cerebral syndrome than females [24]. Recent studies have demonstrated that testosterone and 17β-estradiol differentially regulate mRNA expression of proinflammatory cytokines in the blood and brain during PbA infection [25,26].

Brain swelling caused by vasogenic edema is the most characteristic imaging feature of experimental and human CM and is related to its severity [27–29]. This feature, first observed *in vivo* by magnetic resonance imaging (MRI) in ECM [27] in female CBA/J mice was later confirmed in pediatric CM [6,28]. Other typical MRI findings are parenchymal lesions mostly in or around the white-matter (WM) and hemorrhages which result from blood brain barrier (BBB) disruption [30] and cause a lasting pro-inflammatory state and impaired neuronal function [31].

Sexual dimorphism in susceptibility and immune response to pathogens has been demonstrated in many infectious diseases, with an immunologic advantage for women. In this study, we investigated the hypothesis that sex-differences in the regulation of proinflammatory cytokines previously described in ECM could translate into regional sex-specific differences in the development of vasogenic edema and associated lesions. We examined the impact of biological sex on brain damage caused by ECM in the C57BL/6 mouse strain, in which both sexes are susceptible to CM [19], by quantitatively assessing biomarkers of the cerebral syndrome established or confirmed by MRI (i.e., brain swelling, parenchymal lesions and microhemorrhages). Our results reveal structures commonly affected in both sexes but also the existence of sex-specific lesions in particular structures.

## Materials and methods

### Ethic statement

Animal studies were in agreement with the 2010/63/EU directive of the European Parliament and of the Council of 22 September 2010 and French legislation, i.e., the decree n° 2013−118 of February 1^rst^ and its orders. The project was approved by our institutional committee on ethics (Comité d’Ethique de Marseille n°14) and authorized by the Ministry of Higher Education, Research, and Innovation (project authorization APAFIS #34961).

### Animals

Specific pathogen-free C57BL/6J mice aged 6–8 weeks purchased from Janvier Labs (Le Genest-Saint-Isle, France) were used. The animals were maintained at a temperature of 22−24°C and humidity of ca 50% with 12h light/12h dark cycle in an enriched environment with irradiated bedding material (SAFE BK8-15-10) and free access to food (standard maintenance diet, SAFE A04, SAFE-lab, Rosenberg, Germany) and water. Health monitoring in the animal house facility was performed according to the recommendations using sentinel animals [32].

### Murine model of experimental cerebral malaria

Blinding could not be applied because the same experimenters performed the inoculation of the parasite, the MRI experiments, and data processing. Forty-six C57Bl/6J mice aged 8–10 weeks (19 females and 27 males) were injected with 2 x 10^6^ red blood cells parasitized with PbA *via* intraperitoneal (i.p.) injection. The number of animals per group was calculated based on the effect size for the global increase in brain volume obtained from a pilot study using a level of statistical significance α of 0.05 and a statistical power of 0.8. The group size was 18 animals per sex. This number was increased to consider several risks such as the development of uncomplicated malaria instead of CM in a small percentage of subjects, premature euthanasia if a humane endpoint had been reached or unusable images in case of movement artifacts. The number of males was higher than that of females because the pilot experiment had shown that ECM was more severe in males than females requiring more frequently euthanasia before any MRI exploration could be carried out. The total number of mice was spread over 5 successive experiments. The animals were imaged one week before inoculation of the parasite and at the peak of the cerebral syndrome on day 5–6 post inoculation (p.i.), before they reached the moribund stage. The daily follow-up of the animals consisted in screening for clinical (lethargy, prostration, piloerection, weight loss) and neurological manifestations (ataxia, convulsions, hypothermia, coma). Parasitemia was determined on days 3 and 5 p.i. by Giemsa-stained blood smears. Once the neurological signs of the cerebral syndrome were overt, the animals underwent cerebral exploration using MRI. Animals were euthanized under isoflurane anesthesia at the end of the follow-up or earlier if a humane endpoint had been reached. PbA was a generous gift of Pr. Georges Grau (University of Sydney, Australia).

### *In vivo* MRI protocol

The experiments were conducted on a Bruker AVANCE 500 WB operating at 11.75T (Bruker, Ettlingen, Germany) equipped with actively shielded gradients (1 T/m maximum gradient strength and 9 kT/m/s maximum slew rate) and a transmit and receive birdcage coil for the mouse head (diameter 20 mm). The animals were anesthetized with isoflurane (3%/air at induction and 1–2%/air during the acquisition) and installed in a cradle equipped with the brain coil, a respiratory mask for anesthesia, a pneumatic respiratory probe and a rectal thermometer (thermistor coupled to an optic fiber) for physiological monitoring. The cornea was protected with Ocry-Gel (TVM, Lempdes, France) before placing the animal’s head into the coil. Real-time physiological monitoring was performed using the MR compatible PC-SAM system for mice (Small Animal Instruments, Stony Brook, NY). The body temperature of the mice was kept around 36–37°C during the imaging protocol by using a warm water blanket. Breathing rates were in the range of 90–120 breaths per minute. Anatomical MRI was performed using Bruker Paravision 5.1 and the rapid acquisition with relaxation enhancement (RARE) sequence (RARE factor = 8, time of repetition (TR) = 5000 ms, time of echo (TE) = 9.2 ms, effective TE = 36 ms, number of averages (NA) = 4, 15 x 15 mm^2^ field of view (FOV), 194 x 194 matrix, 31 contiguous axial slices covering the whole brain, 0.5 mm slice thickness).

### MR data processing

The images were segmented using the nnU-Net framework [33] and a training database of eighteen 2D mouse brain MRI acquired at 11.75T and manually segmented by experts at CRMBM. The automatic segmentation produced 28 parcels including the brain mask. All the masks were assessed individually and manually corrected if necessary. The volume of each segmented parcel was calculated as the sum of the area of the structure in all the slices it appears, multiplied by the slice thickness.

For each mouse, the volume fraction (F_V_) of each structure with respect to the whole brain volume was calculated as: $𝐅𝐕={\;𝐕}_{str}/\;{𝐕}_{brain.ctrl}$

V_str_ is the volume of the structure at each time point and V_brain.ctrl_ the volume of the whole brain before disease induction.

$\Delta =\;[({𝐕}_{str.peak}\;-\;{𝐕}_{str.ctrl})/\;{𝐕}_{str.ctrl}]\;*\;100$

V_str.peak_ is the volume of the structure at the peak of the disease and V_str.ctrl_ the volume of the structure before disease induction. The percentage variation of a group of mice for a given structure was obtained by calculating the average Δ.

#### Generation of 3D view of the cortex volume.

Cortical structures were extracted from segmentation results into dedicated NIfTI files using Python (3.9) and NiBabel (5.3.2). 3D meshes were then generated as STL files using 3D Slicer (5.6.1), with a smoothing factor of 0.5, and manually aligned using MeshLab (2023.12). Finally, ParaView (5.13.3) was used to create animations and screen captures.

Views of the brain structures inserted in figures on volumetric results were created using The Scalable Brain Atlas [34,35].

In addition, the images were reviewed to search for characteristic lesions (i.e., hemorrhages, parenchymal lesions) [27,36]. A lesion was considered a microhemorrhage when it appeared as a hypointense well-delineated spot on T_2_-weighted MRI, in either a round or an elongated shape. These were counted for each brain structure, trying to account for those that were continuous along more than one brain slice to avoid duplicate counts. The parenchymal lesions appeared as diffuse or focal hyperintensities and were scored with a yes/no system, noting the presence or absence of them within each structure when compared to the MRI images before the disease.

### Statistical analysis

The statistical analysis was performed using GraphPad Prism 8.0.1, using a Linear Mixed Model analysis with Geisser-Greenhouse correction for the analysis of clinical data, followed by Šidák’s multiple comparison test to assess the evolution of body weight and temperature within a same group, a Log-rank (Mantel-Cox) test for the survival analysis and two-way ANOVAs followed by Šidák’s multiple comparison for the volumetry data. The variables defined for the two-way ANOVA analysis were sex and disease. Their interaction was also tested. Outlying values were not removed from the analysis as they came from correctly segmented artifact-free images. Normality was tested using the Shapiro-Wilk test. Multivariate analysis was performed using JMP® 18.2.0. SAS Institute Inc., and nonparametric Spearman correlation. Values are reported as mean ± SD. The significance level was set at p = 0.05.

## Results

### Clinical follow-up

Ninety-three percent of the males and seventy-four percent of the females inoculated with PbA developed CM (14/19 females and 25/27 males). Animals were sacrificed as they reached pre-established humane end points or at the end of the follow-up (day 7).

**Fig 1:**
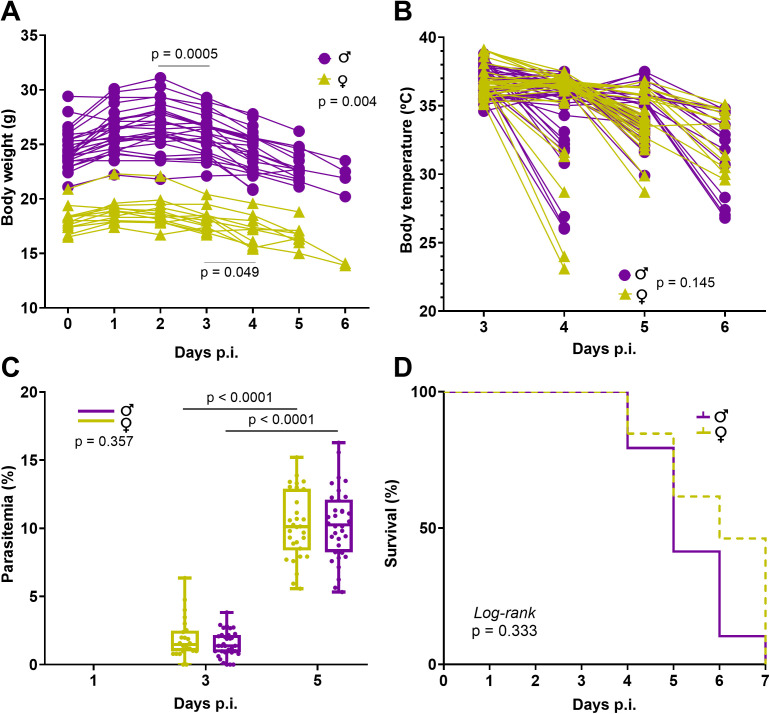
Monitoring of the clinical signs of ECM. ECM is characterized by weight loss (A) that appears earlier in males than in females (at day 3 p.i., p = 0.0005), hypothermia (B), and parasitemia (C). The survival plot (D) includes only mice that developed ECM (14 females and 25 males). Mice were euthanized when under hypothermia (≤ 32°C) with severe clinical signs but are counted as alive in the survival plot on the day of rectal temperature measurement. Boxplots indicate median, min and max. Abbreviations: p.i., post inoculation.

The first clinical manifestation, weight loss, appeared from day 2, while the neurological signs pathognomonic of the severe stage were observed between day 5 and 6 p.i. We observed paralysis of the hind legs exclusively in females (n = 6). The weight loss started at day 2 p.i. for the males (p < 0.001) and at day 3 p.i. for the females (p = 0.001) and progressed until the end of the follow-up (Fig 1A). The dynamics of weight loss differed between the sexes, with a significant interaction between time and sex (p = 0.004). Males lost on average 12% of their body weight (3.2 ± 1.1 g, p < 0.001) and females 14% (2.7 ± 0.8 g, p < 0.001) by day 6 p.i.

Both male and female mice presented significant temperature loss during the cerebral syndrome (p < 0.001). Hypothermia is a well-known characteristic of ECM described in all mouse strains susceptible to the cerebral syndrome [19,37]. In this study, a temperature of 32°C together with severe neurological manifestations served as a criterion to terminate the experiment (Fig 1B). As ECM worsens very rapidly, sudden drops in temperature have occurred overnight in some animals below this value. Body temperature in ECM mice decreased on average by 13% (females 4.8 ± 3.0°C, p < 0.001, males 5.1 ± 2.4°C, p < 0.001) at day 5–6 p.i., with no significant differences between sexes (p = 0.145).

Parasitemia at day 5 p.i. was between 5 and 16% and significantly increased compared to day 3 p.i. (p < 0.001) (Fig 1C). Contrary to what was shown in CBA/Ca mice [24,38], but in agreement with results obtained in FVB/NJ mice [39], no sex difference in parasitemia was detected (p = 0.357) (Fig 1C). There was no difference in survival either (p = 0.333) (Fig 1C, D).

### Description of the brain lesions

Two females and twelve males reached a humane endpoint and had to be sacrificed before being scanned. One male mouse had non exploitable images at both time points. Two females and two males had uncomplete sets of acquisitions with control images that could not be used. In total, ten males and ten females had complete data sets at both follow-up times, and a total of twelve acquisitions could be analyzed for each sex at the peak of CM. Only subjects with complete data sets were analyzed for the volumetric study on edema, whereas all acquisitions available at the peak of CM were analyzed for parenchymal lesions and hemorrhages.

### Hemorrhages

**Fig 2:**
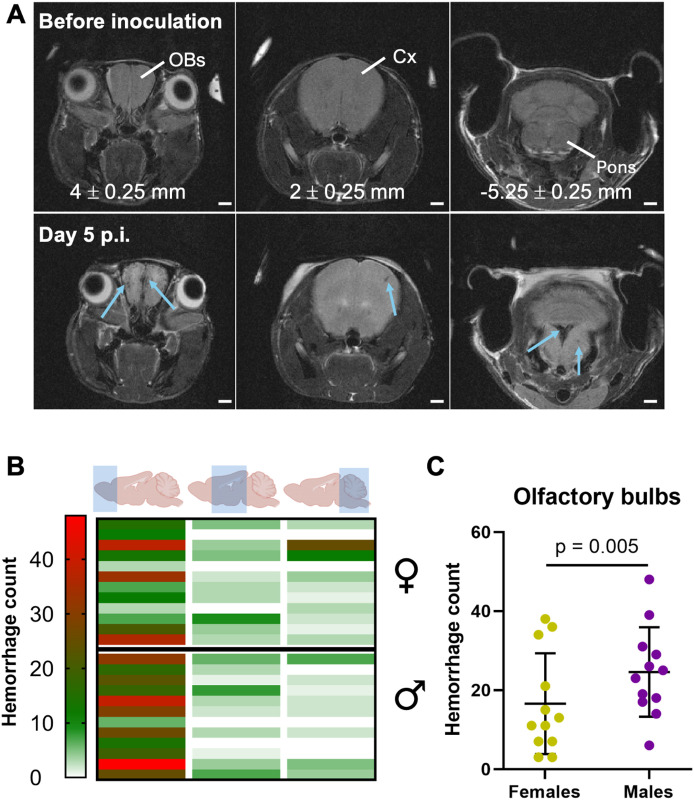
Brain microhemorrhages in ECM. (A) T_2_-weighted images of an anterior, central and posterior region of the brain, from left to right. The top row corresponds to brain images acquired 7 days before the inoculation of PbA and the second row to the corresponding images obtained on day 5 p.i. Values in millimeters indicate approximate distances from bregma and account for the inclination of the coronal MRI plane with respect to the atlas [40]. The bottom row shows best matching MRI slices. Microhemorrhages appear as focal hypointense spots (blue arrows). (B) Number of hemorrhages found per animal in the rostral, central and posterior parts of the brain. Males and females tend to present more hemorrhages in the rostral part, whereas in females they can also be found more frequently in the posterior region as can be seen on the axial MRI showing the pons on day 5 p.i. (C) Males present a significantly higher number of microhemorrhages in the OBs than females (p = 0.005). Each row in B represents one mouse (12 F, 12 M). Data in C are shown as mean ± SD. Abbreviations: Cx, cortex; OBs, olfactory bulbs; p.i., post inoculation. Scale bars: A, 1 mm.

The total number of microhemorrhages was greater in males (29.83 ± 14.22) than in females (27.70 ± 17.88) although not reaching statistical significance (p = 0.758). The hemorrhages occurred throughout the brain, most often in the olfactory bulbs (OBs), and to a lesser extent in the pons and the cortex (Fig 2A, B). Males presented a significantly higher number of hemorrhages in the olfactory bulbs than females (p = 0.005, Fig 2C). In the posterior part of the brain, hemorrhages were mostly found in the medulla oblongata.

### Parenchymal lesions

**Fig 3:**
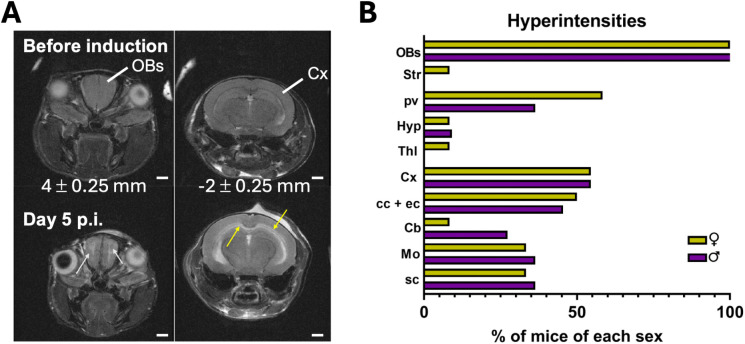
Distribution of parenchymal lesions in ECM by sex. (A) T_2_-weighted images of the brain of the same male mouse acquired before PbA inoculation (top row) and at day 5 p.i. (bottom row). Two images at different anatomical levels are presented showing diffuse hyperintensities in the anterior commissure of the olfactory bulbs (white arrows) and around the corpus callosum and external capsule (yellow arrows). Values in millimeters indicate approximate distances from bregma of the MRI planes. (B) Percentage of animals per sex with lesions in specific brain structures (12 F, 12 M). Abbreviations: Cb, cerebellum; cc + ec, corpus callosum and external capsule; Cx, cortex; Hyp, hypothalamus; Mo, medulla oblongata; OBs, olfactory bulbs; pv, periventricular parenchyma; sc, spinal cord; Str, striatum; Thl, thalamus. Scale bars: A, 1 mm.

At the peak of the cerebral syndrome, all mice of either sex presented hyperintensities in or surrounding the anterior commissure (ac) of the OBs in the region corresponding to the rostral migratory stream (RMS) (Fig 3A). Other hyperintensities were predominantly located in or around the corpus callosum (cc) and the external capsule (ec) (Fig 3B), in the periventricular parenchyma, this latter being more affected in females, in the medulla oblongata and in the spinal cord. Two mice, one female and one male, showed lesions in the hypothalamus. One female mouse presented hyperintensities in the striatum and the thalamus, and 3 males hyperintensities in the cerebellum (Cb). The differences were not significant.

### Volumetric changes

**Fig 4:**
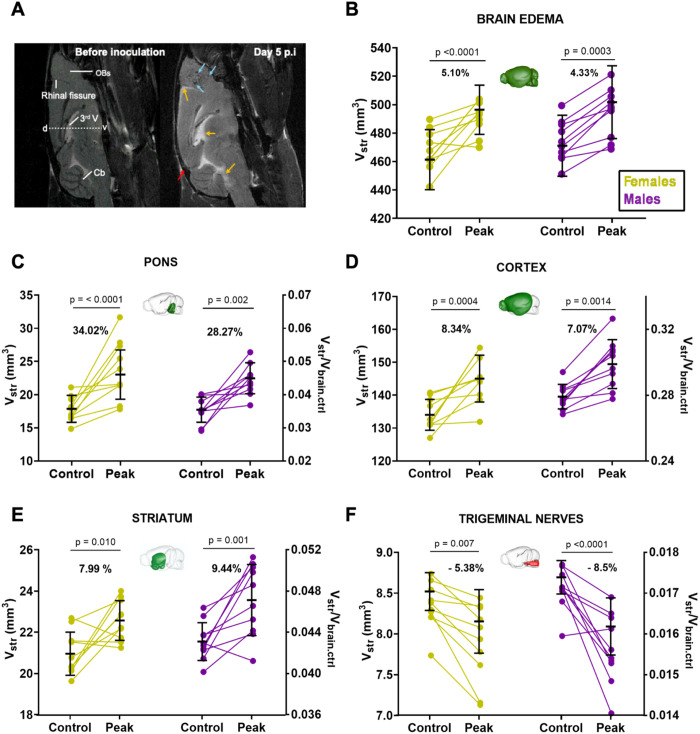
Sex-independent volume changes in ECM. (A) T_2_-weighted sagittal images of the brain of the same male mouse acquired before PbA inoculation and at day 5 p.i. The swelling of the brain leads to a reduction of the intracranial space well visible at the junction between the cerebellum and the inferior colliculus (red arrow). The image on day 5 p.i. shows multiple diffuse hyperintense areas (yellow arrows) and the heavy burden of microhemorrhages in the OBs (blue arrows). (B) At the peak of the cerebral syndrome there is brain swelling in both sexes. Among the brain structures that expand, the pons (C), the cortex (D) and the striatum (E) show a sex independent increase. Contrarily, the cranial part of the trigeminal nerves is subject to crushing or compression in both sexes (F). The volumes are given in mm^3^ (left y-axis) or as volume fraction FV (right y-axis). The conversion to F_V_ was performed by dividing the volumes by 500 mm^3^ which is the approximate brain volume of healthy mice. F_V_ calculated with individual V_brain.ctrl_ are provided in supplementary data. The color code used in the brain views inserted in the graphs indicates whether the brain structure represented is increasing (green), decreasing (red) or remaining unchanged (blue). The statistical analysis was performed using values in mm^3^. Abbreviations: Cb, cerebellum; d, dorsal; OBs, olfactory bulbs; v, ventral; V, ventricle. Data are presented as mean ± SD. 10 males (purple symbols), 10 females (yellow symbols). Scale bar in A: 1 mm.

**Fig 5:**
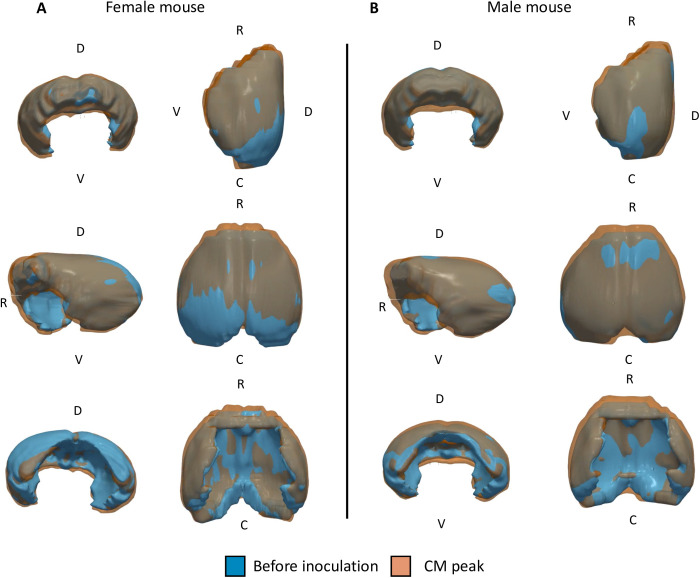
3D-representation of the cortical volume before and during ECM in representative male and female mice. The development of the edema is more advanced rostro-caudally in the female (A) than in the male mouse (B). In both cases, an increase in cortex thickness is observed. This is particularly well visible in the videos of the female mouse (S4 and S5 Videos). Abbreviations: C, caudal; D, dorsal; V, ventral; R, rostral.

Since several approaches are possible to estimate volumetric changes, we expressed them in mm^3^ (Figs 4 and 5) and as volume fractions (S1 and S2 Tables, S3 Fig).

### Brain swelling

At the peak of the cerebral syndrome, both sexes presented significant brain swelling particularly visible along the dorsoventral axis and at the level of the Cb whose fissures are less distinguishable (Fig 4A). The accumulation of fluid causing brain edema is also visible at the level of the rhinal fissure and in the ventricles (Fig 4A)*.* The total brain volume increased by 4–5% at disease peak (males: V_ctrl _= 474.2 ± 14.4 mm^3^, V_peak _= 494.7 ± 17.6 mm^3^; females: V_ctrl _= 467.7 ± 14.2 mm^3^, V_peak _= 491.2 ± 11.6 mm^3^) (Fig 4B). When analyzing the impact of brain edema on each of the 27 substructures individually (S1 and S2 Tables), we noticed that some volume changes were independent of sex whereas others were specific to one sex. Among the 27 brains structures analyzed, 11 showed significant volume changes during CM.

### Impact of edema on the volume of brain structures: Sex-independent changes

Both males and females presented significant augmentation of the volume of the pons (Fig 4C), cortex (Fig 4D) and striatum (Fig 4E). In all three structures the percentage of increase was superior to that of the brain volume increase. Although the pons presented the highest percentage of volume increase, it contributed less to the swelling of the brain than the cortex which was responsible for *ca* 50% of the increase (average cortical increase of 9.9 ± 5.6 mm^3^ in males and 11.0 ± 8.8 mm^3^ in females), while the brain increased 20.5 ± 17.3 mm^3^ in females and 23.5 ± 8.8 mm^3^ in males). Both sexes also presented reductions in the trigeminal nerves’ volumes (Fig 4F). The analysis of volume fractions (S2 Table and S3 Fig) confirmed these results. A 3D representation of the cortex generated from the segmentation masks shows the volume increase caused by CM in one mouse of each sex (Fig 5, S4–S7 Videos). Despite the limitations of manual registration, this representation shows an expansion of the cortex in the rostro-caudal direction and an increase in cortex thickness particularly visible in the videos of the female subject (S4 and S5 Videos). This representation shows that edema spreading is more advanced in the rostro-caudal direction in the female than in the male mouse.

### Impact of edema on the volume of brain structures: sex-specific changes

**Fig 6:**
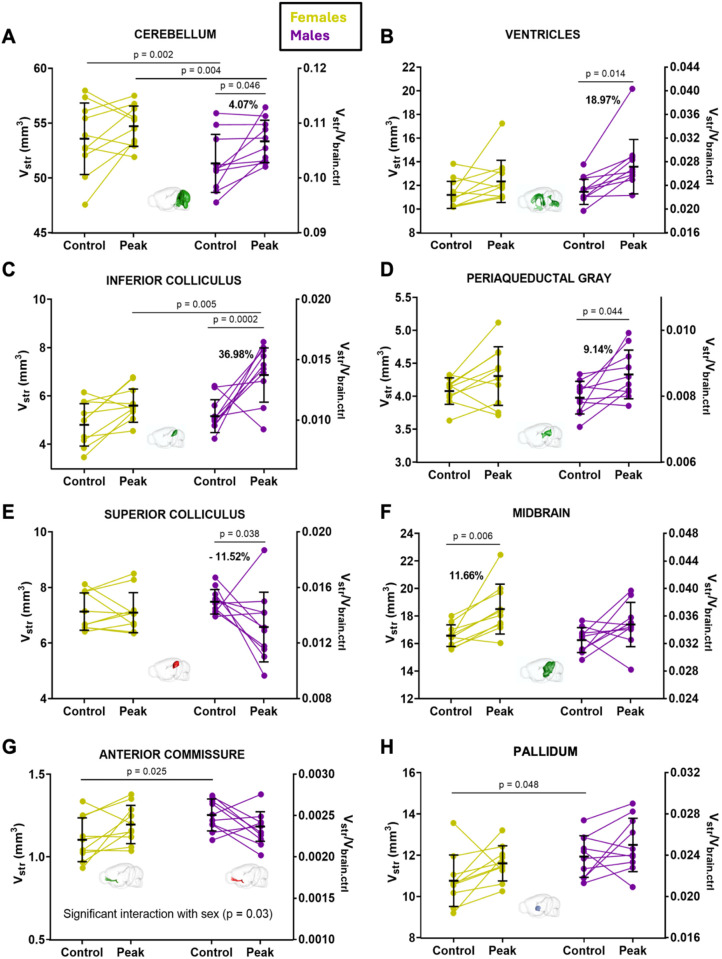
Sex-specific brain volume changes in ECM. Males present increases in the volume of the cerebellum (A), the ventricles (B), the inferior colliculus (C) and the periaqueductal gray (D), and reductions in the superior colliculus (E). The total volume of midbrain (F) significantly augments in the female brain during CM. There is a significant interaction between the sex of the animal and the volume change of the anterior commissure (G). Males and females present differences in the volume of the cerebellum (A), the anterior commissure (G) and the pallidum (H) before disease induction. The volumes are given in mm^3^ (left y-axis) or as volume fraction FV (right y-axis). The conversion to FV was performed by dividing the volumes by 500 mm^3^ which is the approximate brain volume of healthy mice. F_V_ calculated with individual V_brain.ctrl_ are provided in supplementary data (S1, S2 Tables and S3 Fig). The color code used in the brain views inserted in the graphs indicates whether the brain structure represented is increasing (green), decreasing (red) or remaining unchanged (blue). Data are presented as mean ± SD. 10 males (purple symbols), 10 females (yellow symbols). Only the p values corresponding to the comparison of whole groups are shown in this figure. The statistical analysis was performed using values in mm^3^. The p values corresponding to the analysis of subgroups showing opposite behaviors (swelling or shrinkage) for specific brain structures (i.e., ventricles, cerebellum and anterior commissure) are not shown in this figure.

Males presented a significant augmentation of the Cb (Fig 6A), ventricles (Fig 6B), the inferior colliculus (Fig 6C) and the periaqueductal gray (Fig 6D), and a reduction of the superior colliculus (Fig 6E) that was not present or not as pronounced in females. Females presented a significant increase in the volume of the midbrain (Fig 6F). One structure, the ac (Fig 6G), changed volume in a direction that was sex-specific, as it increased in females and decreased in males. The analysis of volume fractions (S2 Table and S3 Fig) confirmed all these results except the one on the ac. Moreover, the olfactory tubercle appeared increased in male mice when analyzing volume fractions (S2 Table).

Additional analyses were carried out, taking account of the different tendencies of volume changes within a group. These analyses were performed for structures known to undergo successively swelling and shrinkage with the progression of CM: Cb (reduced in 4 females, Δ_reduction_ = −1.59 ± 0.79 mm^3^, Δ_variation_ = −2.81%, p = 0.028; augmented in 6 females, Δ_increase_ = 2.96 ± 1.69 mm^3^, Δ_variation_ = 5.84%, p = 0.008), the ventricles (reduced in 3 females, Δ_reduction_ = −0.68 ± 0.27 mm^3^, Δ_variation_ = −5.34%, p = 0.048; augmented in 7 females, Δ_increase_ = 1.99 ± 1.81 mm^3^, Δ_variation_ = 18.12%, p = 0.027). Another structure, the ac in males, was also analyzed because a pattern of evolution similar to that of Cb and ventricles was observed (reduced in 6 males, Δ_reduction_ = −0.18 ± 0.04 mm^3^, Δ_variation_ = −14.12%, p = 0.0001; augmented in 4 males, Δ_increase_ = 0.07 ± 0.05 mm^3^, Δ_variation_ = 6.11%, p = 0.0597).

### Assessment of CM severity using a scale based on MRI biomarkers

**Fig 7:**
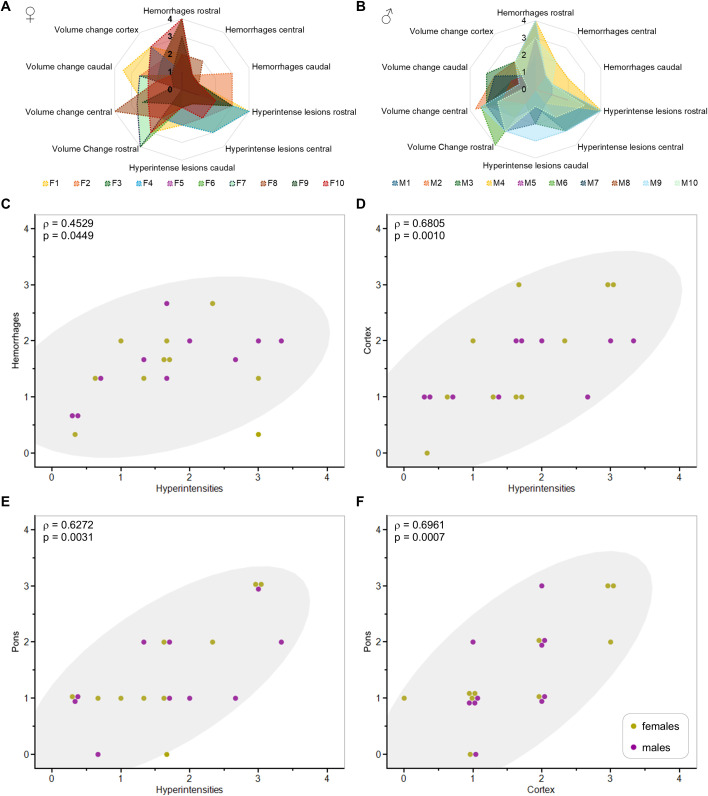
Severity scores based on lesion load and volume change. (A) and (B): radar charts showing severity scores for female (n = 10) and male (n = 10) mice for each of the three MRI biomarkers (hemorrhages, hyperintense lesions and volume changes) in the rostral, central and caudal parts of the brain and in the whole cortex. (C-F): Spearman correlations between the different severity scores obtained for male (purple dots) and female mice (yellow dots) combined (n = 20). (C): correlation between the severity scores for hyperintense and hemorrhagic lesions averaged over the three brain parts. (D): correlation between the average severity score for hyperintense lesions and the severity score for volume changes in cortex. (E): correlation between the average severity score for hyperintense lesions and the severity score for volume changes in pons. (F): correlation between severity scores for volume changes in cortex and pons. Density ellipses with 95% coverage are shown. Full multivariate analysis is shown in S10 Fig.

A severity score has been developed based on our results on MRI biomarkers (hemorrhages, hyperintense lesions and volume changes) (Table 1, Fig 7). This score takes into account the contribution of each region (rostral, central and caudal). Structures anterior to the hippocampus (bregma −0.94 mm) are included in the rostral region, structures posterior to the end of the inferior colliculi (bregma −5.40 mm) are in the caudal region and all structures in between in the central region. Regarding volume changes, only the main structures contributing to brain swelling were considered (rostral region: striatum; central region: midbrain, inferior and superior colliculi; posterior region: pons and cerebellum). The cortex is not associated with a particular region and is considered separately. This score was used to better assess the inter-individual variability in the presentation of CM on anatomical MRI despite the presence of neurological signs characteristic of the cerebral syndrome. Fig 7A, B shows severity scores as radar charts, one chart is presented for each sex (10 mice per group). Individual radar charts are provided in S8 Fig. This score shows that rostral lesions, hemorrhages, and volume changes are prominent severity factors in both groups, together with volume changes in the central and caudal parts of the brain. The cortex volume change, which is the major contributor to global brain swelling, appears more prominent in female than male mice.

**Table 1:** Establishment of a severity score based on MRI biomarkers.

HEMORRHAGES		HYPERINTENSITIES		VOLUME CHANGES	
0	None	0	None	0	None
1	1 to 5]	1	1 structure	1	Mild edema
2	]5–15]	2	2 structures	2	Medium edema
3	]15–30]	3	3 structures	3	Severe edema
4	>30	4	> 3 structures	4	Crushing
**VOLUME CHANGES BY STRUCTURE**					
**CORTEX**	% change	**CEREBELLUM**	% change	**PONS**	% change
0	≤ 1	0	≤ 1	0	≤ 3
1	]1–6]	1	]1–3]	1	]3–30]
2	]6–13]	2	]3–7]	2	]30–60]
3	>13	3	>7	3	>60
4	Crushing	4	Crushing	4	Crushing
**COL SUP**	% change	**COL INF**	% change	**MIDBRAIN**	% change
0	≤ 1	0	≤ 6	0	≤ 3
1	]1–3]	1	]6–20]	1	]3–10]
2	]3–7]	2	]20–40]	2	]10–25]
3	>7	3	> 40	3	>25
4	Crushing	4	Crushing	4	Crushing
**STRIATUM**	% change				
0	≤ 3				
1	]3–9]				
2	]9–15]				
3	>15				
4	Crushing				

Each score was defined on the basis of the range of values obtained in this study. The cumulative severity score is the sum of all scores. A right bracket placed before a number means that this number is excluded, while a right bracket placed after a number means that it is included. Abbreviations: col inf: inferior colliculi, col sup: superior colliculi.

In addition, Spearman correlation analysis for the severity scores obtained for each MRI biomarker was carried out for the structures the most affected by CM (i.e., cortex, pons, inferior and superior colliculi, midbrain, striatum, cerebellum), the three brain regions, and for the severity scores averaged over the three parts of the brain. Spearman correlations were calculated for male and female mice combined (n = 20) as the statistical power was not robust for n = 10. Significant correlations were found between the severity score for hyperintense and hemorrhagic lesions averaged over the three brain regions (Fig 7C), between the average severity score for hyperintense lesions and the severity score for volume changes in cortex (Fig 7D) and pons (Fig 7E), as well as between severity scores for volume changes in cortex and pons (Fig 7F). We observed a significant regional correlation between the severity scores based on lesion load (hemorrhagic and hyperintense lesions). Although within a particular brain region the severity scores for hemorrhagic and hyperintense lesions were not correlated, severity scores for cortex volume change and hyperintense lesions were correlated in each of the three brain regions, suggesting a possible link between cortical swelling and hyperintense lesions. Spearman correlations between regional severity scores for MRI biomarkers are shown in S9 Fig and for each brain structure in S10 Fig.

### Sex differences in brain volumetry before ECM induction

The volume of the whole brain was not significantly different between males and females (p = 0.534) (Fig 4B and S1 Table). Three of the 27 segmented structures showed volumetric differences according to sex. Healthy males have a larger pallidum (p = 0.048, Fig 5H), and ac (p = 0.011, Fig 5G) than females, but a smaller Cb (p = 0.002, Fig 5A). The results on ac and Cb were confirmed by volume fraction analyses (S1 Table).

## Discussion

Parenchyma lesions appearing, independently of sex, as hyperintensities in T_2_-weighted images mainly in the white matter of the olfactory bulbs, corpus callosum and external capsule would result from the combined effects of inflammation, edema and ischemia [27], WM being particularly susceptible to ischemic injury. Histopathological studies in ECM have confirmed myelin injury in the optic nerves and external capsule as well as axonal damage [41,42]. Similar findings have been reported in post-mortem studies on humans [43,44]. Interestingly, the current study reveals that gray matter structures such as the cortex, medulla oblongata, cerebellum and to a lesser extent hypothalamus show hyperintense lesions. These findings are in agreement with studies on adult and pediatric CM reporting cortical and subcortical lesions [45].

The predominance of microhemorrhages in the OBs is in agreement with histological findings [42], and seems related to the thin and leaky structure of the BBB [46,47] predisposing OBs to early inflammatory endothelial damage [48]. Endothelial activation in CM can result in thrombosis [49] and vascular leakage [50] causing hemorrhages. Sex dimorphism in platelet phenotype, coagulation factors and fibrinogen have been demonstrated in humans, with women showing hypercoagulability [51]. In mice, the question is not clear-cut, since both hypercoagulability in C57BL/6N females and higher thrombosis in C57BL/6J males have been described [52,53]. Immunophenotyping has demonstrated sex-specific profiles of resident leucocytes in mice including the C57BL/6 strain [54–56]. Moreover, studies on the effect of sexual hormones in ECM have evidenced higher levels of proinflammatory cytokines TNF and IFN-γ in the OBs of male mice [38]. Consequently, exacerbated inflammation causing vascular damage might be responsible for the higher incidence of microhemorrhages in the OBs of male mice revealed in our study.

Cerebral edema in ECM propagates rostro-caudally *via* the rostral migratory stream (RMS), with the cerebellum and the brainstem being the latest to be affected [48]. In our study, the females had more microhemorrhages than males in posterior structures, which could indicate a greater resistance of females to disease progression, as males generally had to be euthanized earlier than females. Previous studies have revealed brain herniation in mice [27,29] and humans [6,28,57], but only few structures have been studied individually [27,36]. Here, both sexes presented 4–5% global brain volume increase, but strikingly not all structures were affected by vasogenic edema. In both sexes, the percentage of volume increase of the pons, cortex and striatum exceeded that of the whole brain. A few structures exhibited a sex-specific swelling: the ventricles, inferior colliculi and periaqueductal gray in males and the midbrain in females. Although the inferior colliculi and the pons presented the highest relative increase, they contributed less to the global swelling of the brain than the cortex. The swelling of the brain being constrained by the skull, once fissures and cisterns disappear due to the expansion of the outer structures of the cerebrum, the continuous accumulation of vascular fluid in parenchyma may lead to the crushing of inner and posterior structures due to edema propagation along the rostro-caudal axis [48]. The crushing of the cerebellum and compression of the ventricles occur in the severe stage of ECM, while ventricles are enlarged in the mild stage [27]. Decreased ventricle and cerebellum volumes were detected in 3/10 and 4/10 females, respectively, while the males did not reach this stage. These results reflect inter-individual variability in the speed of CM development within a same group [36]. The crushing of the trigeminal nerves, observed in both sexes, is a feature of ECM [36] and is probably caused by a compression exerted by the pons, whereas the crushing of the superior colliculi in males could result from the swelling of the cortex and the cerebellum. Previous studies have suggested that the swelling of the cerebrum is responsible for the crushing of the brainstem in ECM and CM [27,28,36]. Our study demonstrates that the cortex and to a lesser extent pons drive the swelling of the brain in both sexes, and are responsible for the crushing of the medulla oblongata, home to the respiratory and cardiovascular neural centers [58] also involved in body temperature control, leading to coma and death.

We observed sexual dimorphism of the anterior commissure, providing interhemispheric amygdalo-temporal connection, with uninfected males having a larger anterior commissure, as in rats [59], but this remains controversial in humans [60]. After the inoculation of PbA, this dimorphism was present in the form of a sex-dependent response to the infection potentially caused by differences in the regional expression of CD36, a membrane antigen that regulates parasite sequestration and immunity in malaria [61] and that has a higher expression in the anterior commissure of young female mice compared to their male counterparts [62].

Among the possible determinants of edema, regional differences in microvascular density may contribute to the magnitude of fluid accumulation in parenchyma during vasogenic edema. The cortex and subcortical grey matter have a higher density of microvessels than the WM [63], which in the context of endothelial damage would lead to greater water leakage.

Sex hormones are another determinant of edema. Gonadectomy in female mice with ECM reduces survival, suggesting a protective role of estrogens, which downregulate pro-inflammatory cytokines such as TNF and IL-6, while in males, gonadectomy increases survival, consistent with the downregulation of the anti-inflammatory cytokine IL-10 by testosterone [26]. Moreover, the density of androgen receptors in brain is generally higher in male mice, particularly in the OBs [64]. Interestingly, in males with ECM, TNF and IFN-γ expression is superior in the hippocampus, cortex and OBs [38].

Another factor in edema formation could be aquaporin-4, the main water channel in brain, which is involved in the regulation of the extracellular volume and would play a key role in the development of cytotoxic edema and the resolution of vasogenic edema [65]. Immunohistochemistry demonstrated upregulated Aquaporin-4 in ECM [66], while total RNA extraction of the brain revealed a decrease [67]. These discrepancies may be linked to the techniques used but also to the gradients of aquaporin-4 density along rostro-caudal and ventro-dorsal axes in brains of healthy C57BL/6 mice [68]. Interestingly, a study on vasogenic edema demonstrated that estradiol treatment in ovariectomized rats protected their BBB and reduced aquaporin-4 expression in brain [69].

Finally, the mechanical properties of brain structures could also contribute to volume changes of specific structures. Indeed, cortex is stiffer than medulla, brainstem and cerebellum, the latest being the softest structure [70–72].

This study provides new information that could potentially shed light on certain aspects of ECM such as the cerebral origin of hypothermia. Infection can lead to fever and hypothermia [73,74]. Fever is a survival-enhancing adaptative response, whereas hypothermia is regarded as a thermoregulatory failure although some studies suggest that it may be an energy conservation mechanism [73,75,76]. Whether fever or hypothermia is induced would depend on the pathogen load, ambient temperature and species [77,78]. Mild hypothermia may be protective, while deep hypothermia is associated with poor prognosis [79].

ECM is characterized by severe hypothermia unlike human CM where fever is the rule. Periodic fever in human malaria is caused by the schizont rupture of pRBCs [80]. Most of the cytokines causing fever are expressed during CM, the main one being TNF. In severe malaria, the intensity of fever is variable [6,81,82] and precedes loss of consciousness and coma in CM [2]. In two studies on severe malaria and ECM carried out in C57BL/6 mice at thermoneutrality (28°-31°C), hypothermia still occurred and led to similar clinical outcome as observed in mice maintained at sub-thermoneutrality [46,83]. A rise in temperature preceding hypothermia was detected in ECM using a thermal camera [46]. These studies confirm that hypothermia is a key feature of severe malaria and ECM in mice, possibly preceded by a brief episode of fever.

Body temperature is regulated by the preoptic hypothalamus, hypothalamus, medulla oblongata and spinal cord [74,84]. The preoptic hypothalamus (in particular preoptic area and nucleus) which controls fever but also hypothermia in the rodent brain [85,86] is in the rostral part of the brain, which is the first to be affected by edema and lesions in ECM. The hypothalamus was segmented into a single structure because we were unable to identify the boundaries of sub-hypothalamic regions on anatomical MRI. The hypothalamus was neither swollen nor crushed, but in 10% of the animals, hyperintense lesions were detected. Moreover, the preoptic hypothalamus is partly contiguous to the anterior commissure which showed hyperintense lesions in almost all ECM mice. Moreover, medulla oblongata, another structure involved in temperature regulation, showed hemorrhages and hyperintense lesions in almost 40% of the mice. Since the preoptic hypothalamus is the central regulator of fever and hypothermia, we assume that it is probably affected by ECM. Our results suggest that lesions of the medulla oblongata could contribute to temperature dysregulation. We hypothesize that the effect of brain lesions could be amplified by a torpor-like state known to induce hypothermia in mice [85–87] that is controlled by the preoptic hypothalamus and modulated by reduced caloric intake. Thus, the reduction of food intake during ECM which contributes to body weight loss could aggravate hypothermia.

The identification of areas of damage common to males and females as well as sex-specific alterations may advance the understanding of neurological sequelae in CM survivors, that range from cognitive to motor impairments, language deficits or epilepsy, among others [88]. Our results raise the question of whether the outcomes of swelling or crushing for a given structure are different. The immunological, mechanical, and sexual factors outlined above as well as the fact that swelling and compression can occur in the same structure probably determine the severity of tissue damage.

One of the study’s limitations is the absence of additional follow-up points, which would have enabled us to take better account of interindividual variability in the speed of disease progression, as well as to study more precisely the spatial and temporal dynamics of brain swelling. This study was focused on brain swelling, but it is possible that other pathophysiological features of CM such as cytotoxic edema, reduced cerebral blood flow and increased brain lactate may differ according to sex.

## Conclusion

This volumetric study is the first attempt to examine the link between biological sex and brain damage in ECM and to provide a quantification of vasogenic edema and associated lesions throughout the brain. Our results demonstrate for the first time a regionalization of vasogenic edema in CM, with structures affected in both males and females but also sex-specific alterations. Dimorphism in brain lesions evidenced by MRI supports the hypothesis that the modulation of inflammation by sexual hormones previously described in ECM has an impact on the regional development of vasogenic edema as well as the number and distribution of hemorrhages. This knowledge could help improve our understanding of the neurological sequelae of CM and the influence of biological sex, which, if confirmed by further studies, could pave the road to adjunctive therapies aiming at modulating sexual hormones as already considered in other infectious diseases [89].

## Supporting information

S1 Table Volume of the whole brain and of 27 structures before ECM induction. The 2^nd^ and 4^th^ columns correspond to the volume of the brain (V_brain.ctrl_) and of the 27 structures (V_ctrl_) segmented for each sex from images acquired before CM induction. The 3^rd^ and 5^th^ columns correspond to the V_ctrl_/V_brain.ctrl_ ratio. The p values test for the volume difference obtained with volume fractions between males and females (Mann-Whitney test). Values are expressed as mean ± SD.
(DOCX)

S2 Table Volume of the whole brain and of 27 structures at the peak of ECM. The 2^nd^ and 4^th^ columns correspond to the volume of the brain (V_brain.peak_) and of the 27 structures (V_peak_) segmented for each sex from images acquired during CM peak. The 3^rd^ and 5^th^ columns correspond to the V_peak_/V_brain.ctrl_ ratio. The p values in the last column test for the volume difference obtained with volume fractions between males and females (Mann-Whitney test). Values are expressed as mean ± SD.
(DOCX)

S3 Fig Overview of the volume changes of selected brain structures. The volume fractions F_V_ are plotted: V_str.ctrl_/V_brain.ctrl_ before cerebral malaria induction on the x axis, V_str.peak_/V_brain.ctrl_ at the peak of the disease on the y axis. Unaltered volumes appear along the diagonal. Structures remaining within the dotted lines undergo volume changes limited to ± 5%. All 16 selected structures (A). Zoom on selected structures with volume fractions below 0.08 (B), between 0.03 and 0.06 (C), and below 0.034 (D). P values indicate significant F_V_ change with disease in females (yellow) and males (purple). For clarity, small and overlying structures without significant F_V_ change are not displayed.
(TIFF)

S4 Video Animation showing the cortex volume of a female mouse before and during CM. The animation shows the cortex rotating around a ventro-dorsal axis. The mouse is the same as in S5 Video. c, caudal; d, dorsal; v, ventral; r, rostral.
(MP4)

S5 Video Animation showing the cortex volume of a female mouse before and during CM. The animation shows the cortex rotating around rostro-caudal axis. The mouse is the same as in S4 Video. c, caudal; d, dorsal; v, ventral; r, rostral.
(MP4)

S6 Video Animation showing the cortex volume of a male mouse before and during CM. The animation shows the cortex rotating around a ventro-dorsal axis. The mouse is the same as in S7 video. c, caudal; d, dorsal; v, ventral; r, rostral.
(MP4)

S7 Video Animation showing the cortex volume of a male mouse before and during CM. The animation shows the cortex rotating around rostro-caudal axis. The mouse is the same as in S6 video. c, caudal; d, dorsal; v, ventral; r, rostral.
(MP4)

S8 Fig Individual severity scores. Severity scores of each female (A) or male (B) mouse used in the volumetric study (10 males and 10 females). Scores were calculated for each of the three MRI biomarkers (hemorrhages, hyperintense lesions and volume change) in the anterior, central and posterior region of the brain. In addition, a cumulative score including the rostral, central, caudal parts of the brain and the whole cortex is given (the maximum total score is 40). Structures anterior to the hippocampus (bregma −0.94 mm) were included in the “rostral” region, structures posterior to the end of the inferior colliculi (bregma −5.40 mm) were included in the “caudal” region and all structures in between were included in the “central” region.
(TIFF)

S9 Fig Spearman correlations for regional MRI biomarkers. See results section and legend to S8 Fig for definition of rostral, central and caudal regions, (n = 20).
(TIF)

S10 Fig Spearman correlations between severity scores for hemorrhages and hyperintense lesions averaged over the whole brain and for volume changes in each brain structure (n = 20). Abbreviations: Col inf: inferior colliculi, Col sup: superior colliculi.
(TIFF)
