# Supplementary material for: Characterization of experimental cerebral malaria by volumetric MRI A comparative study across the sexes
Source: PLoS One. 2025 Aug 18;20(8):e0328693. doi: 10.1371/journal.pone.0328693 (PMC12360601; doi:10.1371/journal.pone.0328693)
Supplement: S1 Table — The 2nd and 4th columns correspond to the volume of the brain (Vbrain.ctrl) and of the 27 structures (Vctrl) segmented for each sex from images acquired before CM induction. The 3rd and 5th columns correspond to the Vctrl/Vbrain.ctrl ratio. The p values test for the volume difference obtained with volume fractions between males and females (Mann-Whitney test). Values are expressed as mean ± SD. (DOCX) [file pone.0328693.s001.docx]

| STRUCTURE (CTRL) | Brain structure volume (mm^3^)  Female mice | V_ctrl_/V_brain.ctrl_  Female mice | Brain structure volume (mm^3^)  Male mice | V_ctrl_/V_brain.ctrl_  Male mice | p-value |
| --- | --- | --- | --- | --- | --- |
| Total brain | 467.66 ± 14.15 | 1 | 474.21 ± 14.37 | 1 | 0.534 |
| Optic nerves and tracts | 1.93 ± 0.15 | 0.004 ± 0.0003 | 1.98 ± 0.25 | 0.004 ± 0.0005 | 0.971 |
| Trigeminal nerves | 8.34 ± 0.28 | 0.017 ± 0.0003 | 8.56 ± 0.25 | 0.018 ± 0.0009 | 0.752 |
| Anterior commissure | 1.11 ± 0.13 | 0.002 ± 0.0002 | 1.13 ± 0.41 | 0.002 ± 0.0008 | 0.017 |
| Corpus callosum and cingulum | 10.17 ± 0.56 | 0.022 ± 0.0008 | 9.88 ± 0.47 | 0.021 ± 0.0009 | 0.509 |
| Internal capsule | 8.02 ± 0.60 | 0.017 ± 0.001 | 7.71 ± 0.64 | 0.016 ± 0.001 | 0.758 |
| Ventricles | 11.40 ± 1.20 | 0.024 ± 0.002 | 11.68 ± 1.12 | 0.025 ± 0.002 | 0.990 |
| Hippocampus | 25.73 ± 1.04 | 0.055 ± 0.002 | 26.04 ± 0.83 | 0.055 ± 0.0003 | 0.993 |
| Cerebellum | 53.59 ± 3.26 | 0.115 ± 0.004 | 51.34 ± 2.65 | 0.108 ± 0.003 | 0.002 |
| Superior colliculi | 7.17 ± 0.68 | 0.015 ± 0.001 | 7.52 ± 0.45 | 0.016 ± 0.001 | 0.743 |
| Inferior colliculi | 4.87 ± 0.90 | 0.010 ± 0.002 | 5.24 ± 0.70 | 0.011 ± 0.001 | 0.718 |
| Olfactory bulbs | 30.25 ± 1.77 | 0.065 ± 0.005 | 30.83 ± 1.76 | 0.065 ± 0.004 | 0.992 |
| Hypothalamus | 13.04 ± 0.43 | 0.028 ± 0.001 | 13.42 ± 0.51 | 0.028 ± 0.002 | 0.685 |
| Thalamus | 20.74 ± 0.84 | 0.044 ± 0.002 | 21.05 ± 0.90 | 0.044 ± 0.002 | 0.999 |
| Pons | 17.75 ± 1.88 | 0.038 ± 0.004 | 17.58 ± 1.85 | 0.037 ± 0.004 | 0.948 |
| Striatum | 20.98 ± 1.05 | 0.045 ± 0.001 | 21.57 ± 0.92 | 0.045 ± 0.001 | 0.788 |
| Midbrain | 16.58 ± 0.78 | 0.035 ± 0.001 | 16.26 ± 0.90 | 0.034 ± 0.002 | 0.581 |
| Cortex | 134.05 ± 4.69 | 0.286 ± 0.004 | 139.56 ± 3.74 | 0.294 ± 0.004 | 0.289 |
| Vestibulochoclear nerve | 0.34 ± 0.04 | 0.001 ± 9e-05 | 0.31 ± 0.05 | 0.0006 ± 0.0001 | 0.170 |
| Septum | 3.11 ± 0.22 | 0.007 ± 0.0003 | 3.30 ± 0.21 | 0.007 ± 0.0004 | 0.234 |
| Medulla oblongata | 29.82 ± 2.59 | 0.064 ± 0.005 | 30.42 ± 3.32 | 0.064 ± 0.007 | 0.993 |
| Substantia nigra | 1.62 ± 0.23 | 0.003 ± 0.0004 | 1.72 ± 0.36 | 0.004 ± 0.0007 | 0.685 |
| Pallidum | 10.78 ± 1.25 | 0.023 ± 0.002 | 11.95 ± 1.01 | 0.025 ± 0.002 | 0.066 |
| Periaqueductal gray | 4.08 ± 0.20 | 0.009 ± 0.001 | 3.98 ± 0.25 | 0.008 ± 0.0006 | 0.608 |
| Amygdala | 9.53 ± 0.53 | 0.020 ± 0.001 | 9.96 ± 0.52 | 0.021 ± 0.0007 | 0.313 |
| Accumbens nucleus | 1.40 ± 0.21 | 0.003 ± 0.0004 | 1.71 ± 0.53 | 0.004 ± 0.001 | 0.259 |
| Hippocampal commissure | 0.20 ± 0.04 | 0.0004 ± 8e-05 | 0.24 ± 0.02 | 0.0005 ±  4e-05 | 0.010 |
| Olfactory tubercle | 1.47 ± 0.17 | 0.003 ± 0.0003 | 1.57 ± 0.28 | 0.003 ± 0.0006 | 0.267 |
