# Supplementary figures and images for: Characterization of experimental cerebral malaria by volumetric MRI A comparative study across the sexes
Source: PLoS One. 2025 Aug 18;20(8):e0328693. doi: 10.1371/journal.pone.0328693 (PMC12360601; doi:10.1371/journal.pone.0328693)

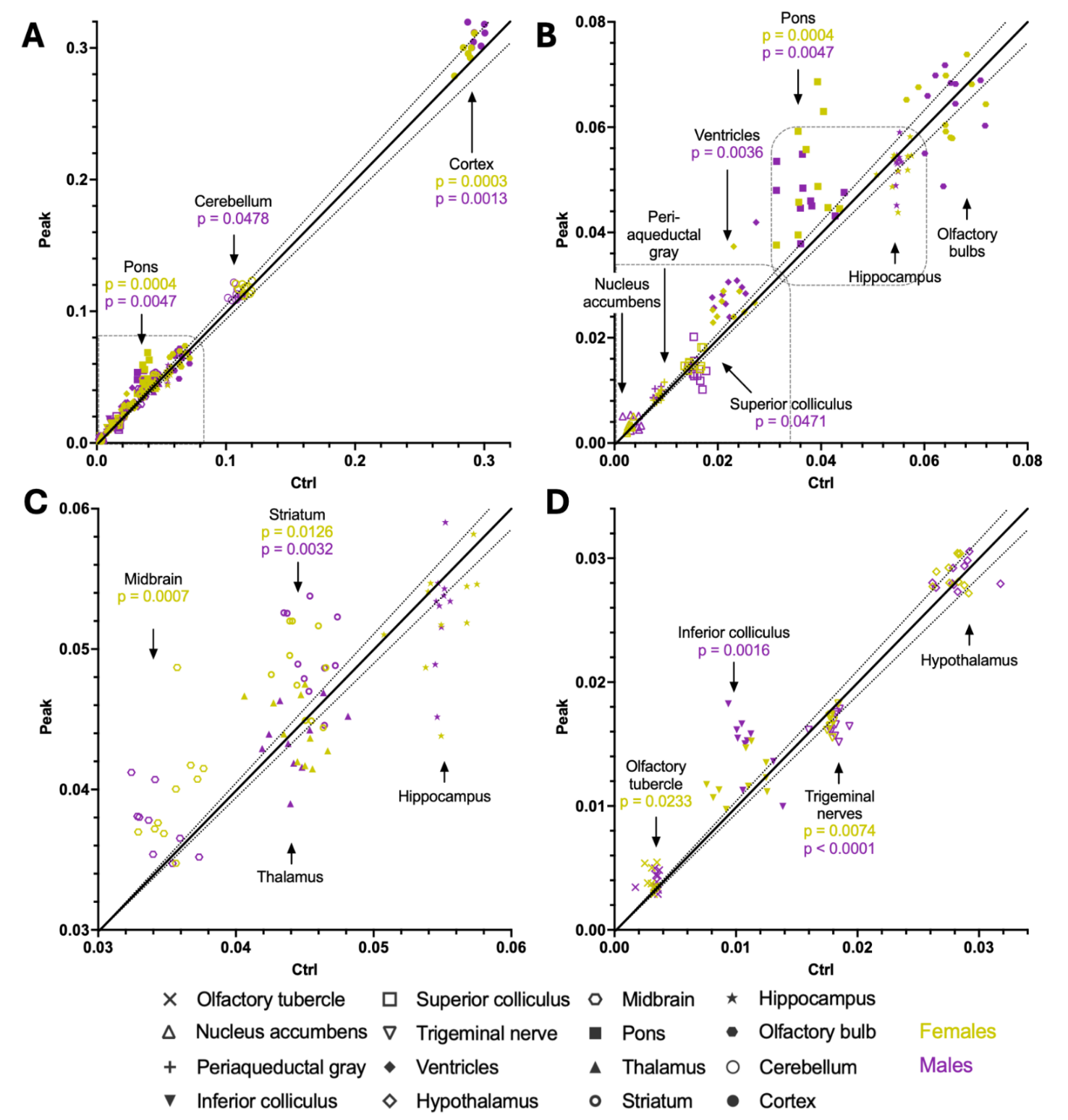

Supplement: S3 Fig — The volume fractions FV are plotted: Vstr.ctrl/Vbrain.ctrl before cerebral malaria induction on the x axis, Vstr.peak/Vbrain.ctrl at the peak of the disease on the y axis. Unaltered volumes appear along the diagonal. Structures remaining within the dotted lines undergo volume changes limited to ± 5%. All 16 selected structures (A). Zoom on selected structures with volume fractions below 0.08 (B), between 0.03 and 0.06 (C), and below 0.034 (D). P values indicate significant FV change with disease in females (yellow) and males (purple). For clarity, small and overlying structures without significant FV change are not displayed. (TIFF) [file pone.0328693.s003.tiff]

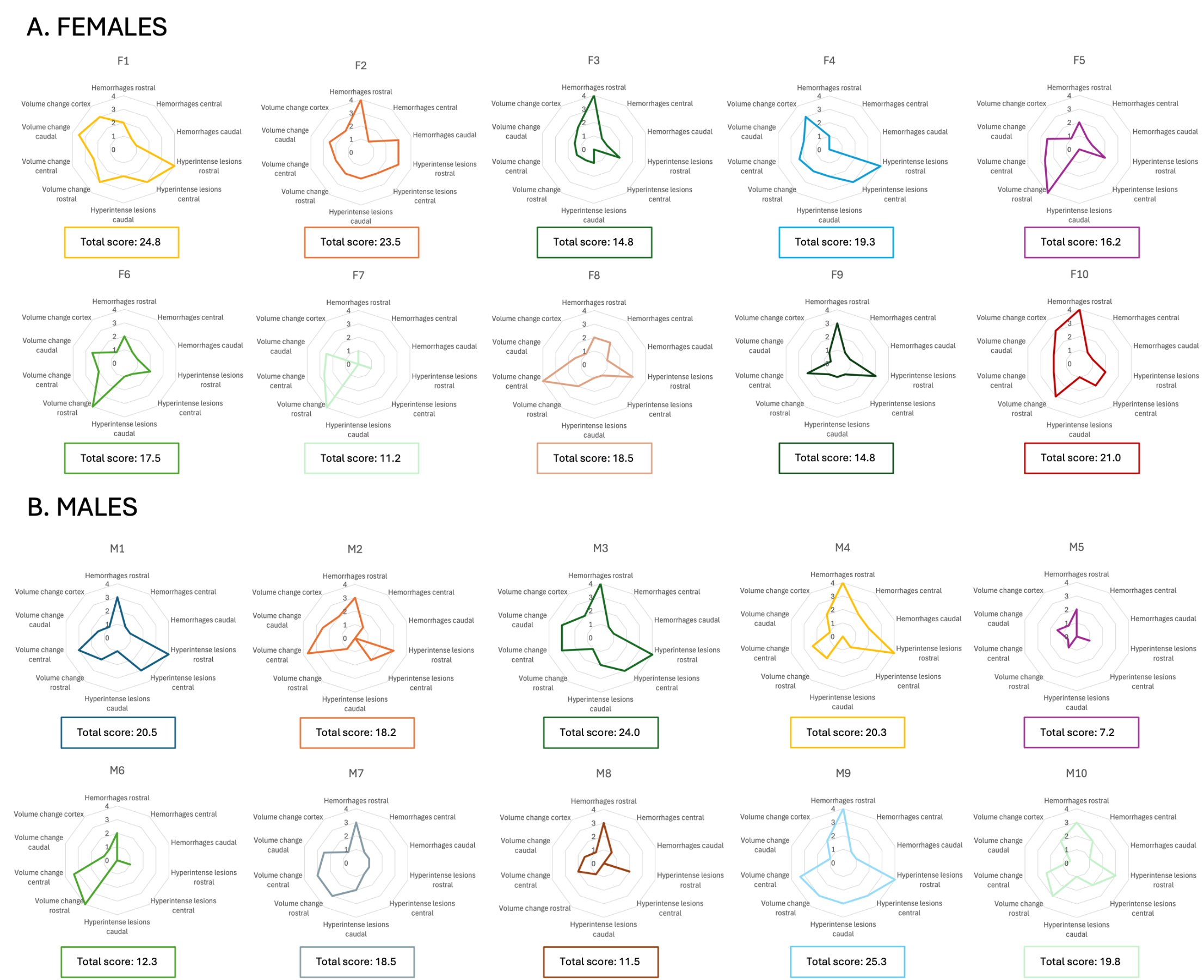

Supplement: S8 Fig — Severity scores of each female (A) or male (B) mouse used in the volumetric study (10 males and 10 females). Scores were calculated for each of the three MRI biomarkers (hemorrhages, hyperintense lesions and volume change) in the anterior, central and posterior region of the brain. In addition, a cumulative score including the rostral, central, caudal parts of the brain and the whole cortex is given (the maximum total score is 40). Structures anterior to the hippocampus (bregma −0.94 mm) were included in the “rostral” region, structures posterior to the end of the inferior colliculi (bregma −5.40 mm) were included in the “caudal” region and all structures in between were included in the “central” region. (TIFF) [file pone.0328693.s008.tiff]

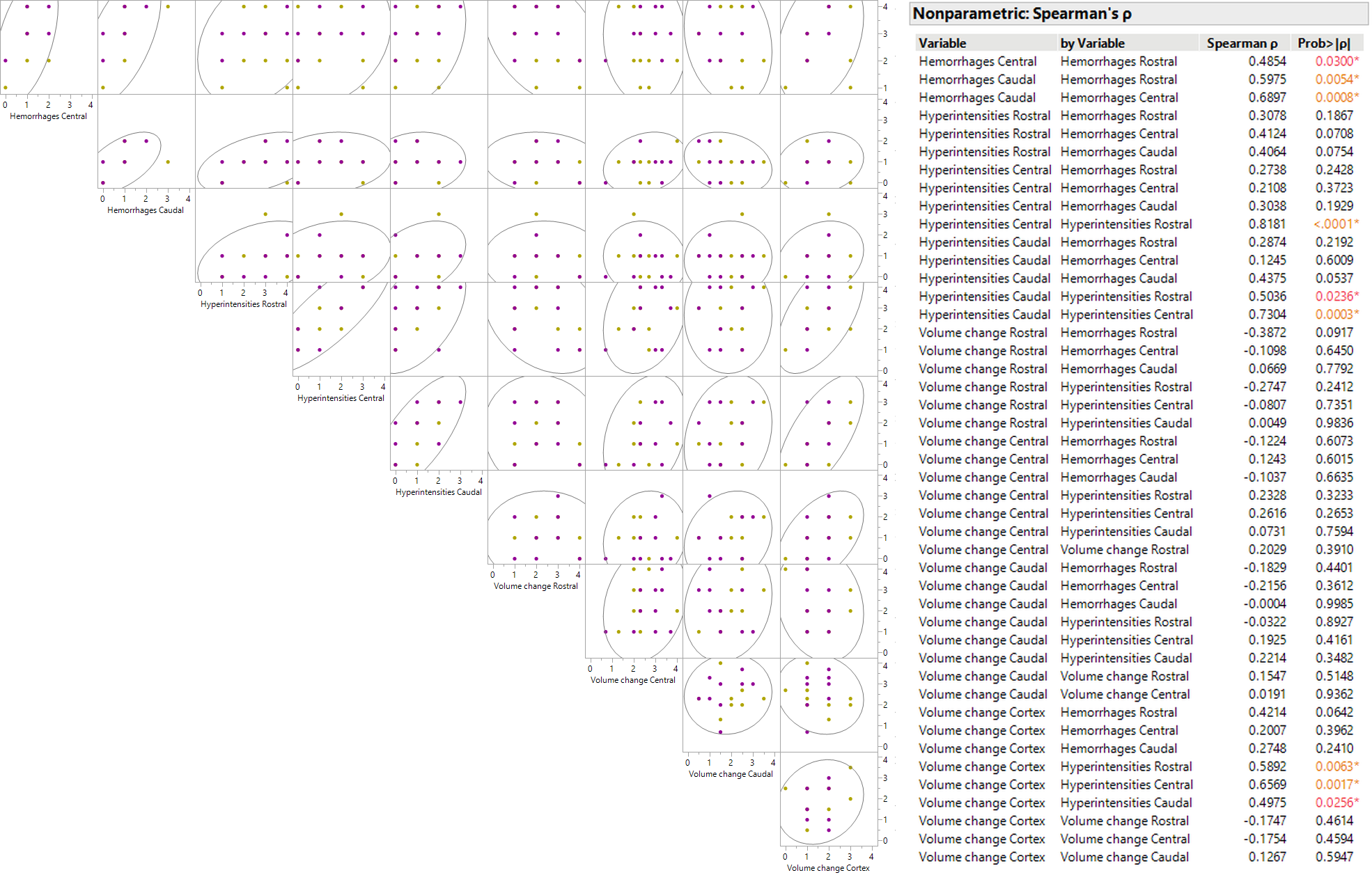

Supplement: S9 Fig — See results section and legend to S8 Fig for definition of rostral, central and caudal regions, (n = 20). (TIF) [file pone.0328693.s009.tif]

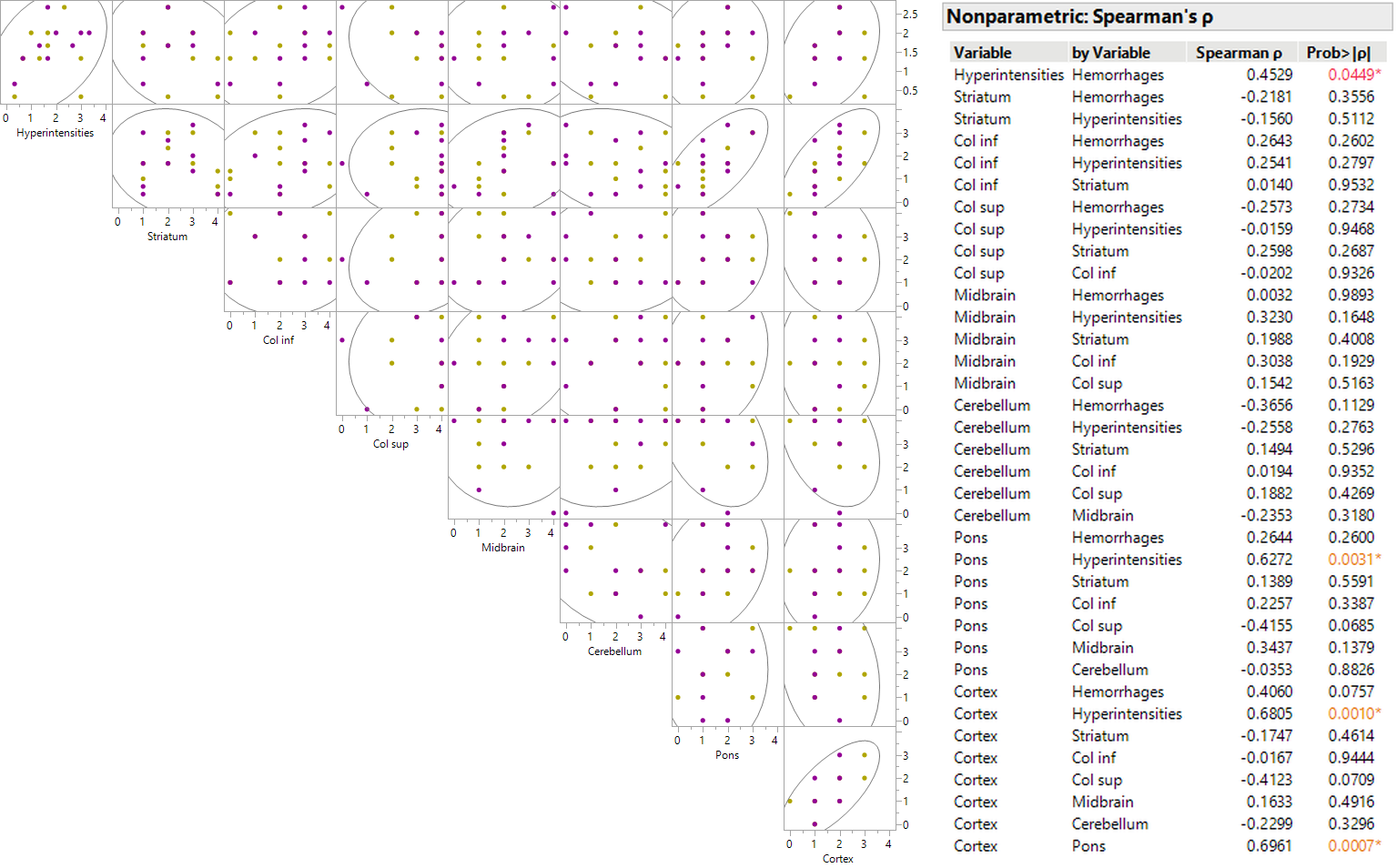

Supplement: S10 Fig — Abbreviations: Col inf: inferior colliculi, Col sup: superior colliculi. (TIFF) [file pone.0328693.s010.tiff]
